# Comparison of McGrath Videolaryngoscope and Macintosh Laryngoscope in Children with Torticollis: Randomized Controlled Trial

**DOI:** 10.3390/children8121171

**Published:** 2021-12-10

**Authors:** Min Hur, Jong Yeop Kim, Sang Kee Min, Kyuheok Lee, Young Ju Won, Ji Eun Kim

**Affiliations:** 1 Department of Anesthesiology and Pain Medicine, Ajou University School of Medicine, Suwon 16499, Korea; nuage1220@gmail.com (M.H.); kjyeop@ajou.ac.kr (J.Y.K.); anesmin@nate.com (S.K.M.); mybfgg@gmail.com (K.L.); 2 Department of Anesthesiology and Pain Medicine, Korea University Guro Hospital, Seoul 08308, Korea

**Keywords:** anesthesia, airway management, intubation, pediatrics, torticollis

## Abstract

We investigated the efficacy of the McGrath videolaryngoscope compared with the Macintosh laryngoscope in children with torticollis. Thirty children aged 1–10 years who underwent surgical release of torticollis were randomly assigned into the McGrath and Macintosh groups. Orotracheal intubation was performed by a skilled anesthesiologist. The primary outcome was the intubation time. The Cormack–Lehane grade, lifting force, intubation difficulty scale (IDS), difficulty level, and intubation failure rate were also assessed. The intubation time was significantly longer in the McGrath group than in the Macintosh group (31.4 ± 6.7 s vs. 26.1 ± 5.4 s, *p* = 0.025). Additionally, the Cormack–Lehane grades were comparable between the groups (*p* = 0.101). The lifting force and IDS were significantly lower in the McGrath group than in the Macintosh group (*p* < 0.001 and *p* = 0.022, respectively). No significant differences were observed with respect to endotracheal intubation difficulty and intubation success rate. Intubation-related complications were also not observed. In conclusion, compared with the Macintosh laryngoscope, the McGrath videolaryngoscope extended the intubation time and did not improve glottic visualization in children with torticollis, despite having a lesser lifting force, lower intubation difficulty scale, and similar success rate.

## 1. Introduction

Congenital muscular torticollis is a common musculoskeletal anomaly with an incidence of up to 16% of healthy newborns [1]. It is characterized by ipsilateral cervical lateral flexion and contralateral cervical rotation caused by unilateral contracture of the sternocleidomastoid muscle [2]. Up to 90% of infants with congenital muscular torticollis also have craniofacial asymmetry as a coexisting impairment [3]. If left untreated, the degree of facial asymmetry or plagiocephaly may progress and result in the development of secondary changes, such as cervical spine dysmorphism and limited neck mobility [4,5,6,7]. Thus, in cases that do not resolve after physical therapy, surgical correction is necessary in earliest childhood to obtain good clinical outcomes [2,7].

The McGrath videolaryngoscope (VL) (McGrath MAC^®^; Aircraft Medical Ltd., Edinburgh, UK) is a commonly used VL that features classically shaped angulated laryngoscopy blades and incorporates a high-resolution camera at the tip of the blade. Although studies on adults have demonstrated that the McGrath VL is superior to the Macintosh laryngoscope to some degree [8], few comparative studies have been conducted in children. A previous study described the usefulness of the McGrath VL in easily intubating 100 children without complications [9]. In addition, the McGrath VL showed greater intubation effectiveness than direct laryngoscopes in pediatric manikins, such as those simulating cardiopulmonary resuscitation, Cormack–Lehane grade IV, and immobilized cervical spine [10,11,12]. In a prospective randomized study, the McGrath VL provided a better glottic view and lower intubation difficulty scale (IDS) than the Macintosh laryngoscope in children with normal airway [13].

Pediatric airway management is a challenge for clinicians because of the unique airway features and rapid oxygen desaturation in cases of failure in securing the airway [14]. Moreover, congenital craniofacial anomalies characterized by dysmorphic features are often associated with difficult intubation [15]. To date, no clinical trials have investigated the usefulness of VLs in children having anomalies that lead to an anticipated difficult airway. To our knowledge, this is the first study to compare the McGrath VL to direct laryngoscopes in children with congenital muscular torticollis. This study aimed to assess the efficacy of the McGrath VL compared with the Macintosh laryngoscope in children with torticollis who require endotracheal intubation for general anesthesia.

## 2. Materials and Methods

### 2.1. Study Design

This prospective randomized study was conducted among children undergoing surgical release of torticollis at the Ajou University Hospital between May 2018 and May 2019. The study was approved by the Institutional Review Board of Ajou University Hospital (protocol number: AJIRB-MED-OBS-18-106, interventional study, 25 May 2018) and was registered at ClinicalTrials.gov (NCT 03582787). The study was conducted according to the Declaration of Helsinki, and all children and their parents provided written informed consent.

### 2.2. Study Population

Children aged 1–10 years with an American Society of Anesthesiologists physical status 1 or 2, who were diagnosed with congenital muscular torticollis and underwent an elective surgical release of torticollis under general anesthesia with orotracheal intubation, were included. The exclusion criteria were a history of difficult airway (failed laryngoscopy or intubation), anticipated difficult airway on preoperative evaluation, congenital anomaly involving the respiratory tract, cervical spine instability, upper respiratory infection within the previous 4 weeks, a history of asthma, neuromuscular diseases, or the need for rapid-sequence intubation.

At our institute, surgical release of congenital muscular torticollis is performed when the children simultaneously meet the following three criteria: age ≥ 6 months, evident functional limitation of neck mobility due to shortening of the unilateral sternocleidomastoid muscle, and parents’ realization that stretching exercises are not beneficial.

### 2.3. Anesthesia and Interventions

Children (*n* = 30) were randomly assigned in a 1:1 ratio to either the McGrath group or the Macintosh group using a computer-based randomization program. The group assignments were sealed in opaque envelopes by a research assistant not involved in the study. On arrival at the operating room, the sealed envelope was opened by the nurse in charge of study preparation who gave the assigned laryngoscope to the laryngoscopist immediately before intubation. The patients, investigators, data analysts, and outcome assessors were also blinded to the group allocation during the study period.

No pre-medication was performed. In the operating room, all children received standard monitoring, including blood pressure measurement, pulse oximetry for measuring oxygen saturation (SpO_2_), and electrocardiography. After pre-oxygenation with 100% oxygen for 1 min, anesthesia was induced using 2.5–3.0 mg/kg of intravenous (IV) propofol and 1 mcg/kg of IV fentanyl. After confirming the absence of a response to an eye stimulus, 1 mg/kg of IV rocuronium was administered. Mask ventilation with 100% oxygen and sevoflurane (3–5 vol%) was delivered to all children. After confirming a zero of train-of-four using a nerve stimulator (TOF-Watch^®^ Organon, Dublin, Ireland), orotracheal intubation was performed by a laryngologist using the McGrath VL or Macintosh laryngoscope according to the group assignment. Thereafter, anesthesia was maintained with sevoflurane (2.5–3.5 vol%).

During orotracheal intubation, children’s heads were placed on a soft donut-shaped foam headrest to support their heads, but a shoulder roll was not used. The cuffed endotracheal tubes were prepared by bending the tip of the tube at an angle of 60° using the stylet [16]. The tube size was calculated according to the following formula: tube size = 3.5 + child’s age/4. In the McGrath group (*n* = 15), laryngoscopy was performed with the tip of the blade placed in the vallecula without displacing the tongue. In the Macintosh group (*n* = 15), laryngoscopy was performed with the tip of the blade also placed in the vallecula, but it was inserted in the right labial commissure of the mouth and the entire tongue was swept to the left side of the blade. The styletted tube in both groups was passed to the right of the blade. An assistant anesthesiologist removed the stylet from the intubation tube when the tip of the endotracheal tube had passed the vocal cords. All laryngoscopies were performed by a single independent anesthesiologist with at least 15 years of intubation experience in general pediatrics, including children with torticollis.

### 2.4. Data Collection

The primary endpoint was the ‘intubation time’, which was defined as the time from insertion of the laryngoscope blade between the teeth or gums until the detection of the first capnography upstroke. Initially, preoperative evaluations, such as the Mallampati class, neck mobility, and loose upper incisors, were planned. However, due to the children’s non-cooperation, the Mallampati class was not determined, and neck mobility was assessed by the laryngologist after inducing anesthesia using propofol and before neuromuscular blockade using rocuronium. The Cormack–Lehane grade was assessed by the laryngologist when the best view of the glottic opening was achieved [17]. The external pressure to the larynx (the optimal external laryngeal manipulation, OELM) and/or increased lifting force was applied at the laryngologist’s discretion to optimize the laryngeal view and facilitate endotracheal intubation. We also assessed the IDS, which is a validated numerical description of the difficulty of intubation based on seven quantitative and qualitative aspects of the procedure (Table 1). In addition, the difficulty of endotracheal intubation was assessed by the laryngologist using a subjective scale (1, easy; 2, moderate; and 3, difficult to intubate). ‘Intubation failure’ was defined as failure to intubate within 60 s or inadequate oxygenation/ventilation (SpO_2_ < 95%) at the first attempt. In case of an ‘intubation failure’, a second intubation was performed, and if failure persisted despite the second trial, an alternative maneuver was considered. After completing surgery, intubation-related complications were evaluated as follows: coughing, laryngospasm, bronchospasm, desaturation (SpO_2_ < 90%), regurgitation, blood stain on the removed blade, and trauma to the lips, tongue, or teeth. Hemodynamics, including the mean blood pressure, heart rate, and SpO_2_, were recorded at four time points: at baseline (before induction), 1 min after induction, before intubation, and 1 min after intubation.

### 2.5. Statistical Analysis

The sample size calculation was based on the primary outcome. In a previous study, the mean ± standard deviation (SD) of the intubation time when neonates with difficulty airway (Cormack–Lehane grades III and IV) were intubated using a direct laryngoscope was 67.2 ± 8.54 s [18]. Assuming a clinically significant increase in intubation time of 10 s or more during intubation using the McGrath VL, 12 patients in each group were required with a type 1 error of 0.05 and a power of 0.8. Considering a dropout rate of approximately 20%, we enrolled a total of 30 patients.

Data are expressed as mean ± SD, median (interquartile range, (range)), or the number of patients (%). The normality of data distribution was tested using the Kolmogorov–Smirnov test. Continuous variables were compared using Student’s *t*-test for normally distributed data or the Mann–Whitney U-test for non-normally distributed data. Dichotomous variables were compared using the chi-square or Fisher’s exact tests, as appropriate. A *p* < 0.05 was considered statistically significant. Data analyses were performed using G*power (version 3.1.9.2, Universität Düsseldorf, Düsseldorf, Germany) and IBM SPSS Statistics for Windows/Macintosh, Version 22.0 (IBM Corp., Armonk, NY, USA).

## 3. Results

### 3.1. Study Population

**Figure 1:**
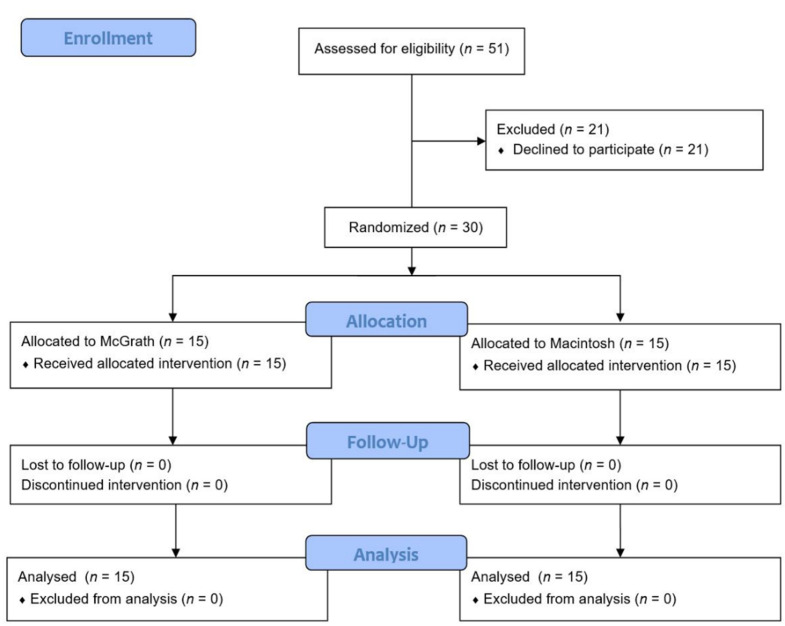
Consort flow diagram of patient enrollment.

A total of 30 patients were enrolled and received the allocated interventions (Figure 1).

### 3.2. Baseline Characteristics and Hemodynamics

Significant differences were not observed in children’s characteristics, surgical findings, and preoperative airway assessment (Table 2). Abnormal neck mobility was found in 18 children, but it did not differ between the two groups (10 children in the McGrath group and 8 children in the Macintosh group). Furthermore, no significant intergroup differences were observed in hemodynamics throughout the study (Table 3).

### 3.3. Intubation Parameters

The intubation time was the primary outcome in this study. It was significantly longer in the McGrath group than in the Macintosh group (31.4 ± 6.7 s vs. 26.1 ± 5.4 s, *p* = 0.025; Table 4). The Cormack–Lehane grade was similar between the two groups (*p* = 0.101), and the need for OELM showed no intergroup difference (*p* = 0.427). However, the lifting force was significantly higher in the Macintosh group than in the McGrath group (*p* < 0.001). Consequently, the IDS was significantly higher in the Macintosh group than in the McGrath group (1 (0–2) vs. 2 (1–3), *p* = 0.022). The difficulty level of endotracheal intubation did not differ between the two groups (*p* > 0.999). Moreover, no significant intergroup difference was observed in the intubation failure rate (*p* > 0.999). Intubation failure occurred in three children because of difficulties in the alignment of the vocal cord, blade, and stylet angle axes. Thus, two children (one each in the two groups) were intubated after the second attempt, and one child in the McGrath group had an intubation time exceeding 60 s.

### 3.4. Adverse Events

None of the blades needed to be exchanged or replaced. During laryngoscopy, episodes of bradycardia or arrhythmias were not noted, and the minimum SpO_2_ values when using the two blades were similar. Intubation-related complications were not observed in either group after endotracheal intubation or during anesthetic emergence.

## 4. Discussion

In this randomized controlled study, the intubation time was significantly extended when using the McGrath VL than when using the Macintosh laryngoscope. In addition, the McGrath VL did not improve glottic visualization, although it required a lesser lifting force and had a lower IDS than the Macintosh laryngoscope. Nevertheless, intubation failure rate or intubation-related complications were similar between the two groups.

Congenital muscular torticollis is characterized by excessive muscle atrophy and fibrosis, leading to tightness of the sternocleidomastoid muscle and limited cervical mobility. Clinically, it is associated with facial deformities and asymmetry [4], including an ipsilateral recessed orbit and zygoma, deviation of the nose and chin, and inferiorly and posteriorly positioned ipsilateral ear. This could lead to discrepancy between the facial midline and cervical midline during intubation. Moreover, plagiocephaly appears simultaneously, and it is known to occur in children younger than 6 months [19]. This induces head rotation to one side when lying supine. This condition is also characterized by apparent anatomical changes at the level of the atlas and axis vertebrae, which exhibit rotational and bending deformities [6]. Such anatomical changes start manifesting at around 8 months of age [5]. These could affect the sniffing position during intubation.

Studies on adult populations have suggested that the McGrath VL allows superior glottic visualization but requires lengthier intubation time than direct laryngoscopes [8]. However, there remains a paucity of data regarding the efficacy of the McGrath VL in pediatric patients. In a meta-analysis including 1196 participants, VLs were found to be a good alternative to direct laryngoscopes during tracheal intubation; however, only 4 of the 11 trials in this meta-analysis involved children, and only one of the adult studies used the McGrath VL [20]. In a meta-analysis conducted in 2014, Sun et al. found that the VLs improved glottic visualization compared to direct laryngoscopes in children with normal airways or those with anticipated difficult airway [21]. However, the study using the McGrath VL was not included in the final analysis. Another study conducted in the operating room compared the McGrath VL with the Macintosh laryngoscope in children, but all the included children had normal airways [13].

In our study, the average intubation time in the McGrath group was significantly longer than that in the Macintosh group (31.4 s vs. 26.1 s). These values are reliable when compared with the intubation times obtained for children with normal airways at the same institute (25 s and 26 s for the McGrath and Macintosh groups, respectively) [13]. However, our results are inconsistent with those of other studies on pediatric manikins simulating difficult intubation. In those studies, the intubation time was similar when performed by anesthesiologists and rather shorter in the McGrath VL than direct laryngoscopes when performed by paramedics [10,11,12]. It is interesting that, in our study, the intubation time was extended in McGrath VL compared to the Macintosh laryngoscope, despite children having torticollis and intubation being performed by an anesthesiologist with >15 years of experience. Meanwhile, our finding is similar to that of a meta-analysis of adults, wherein the McGrath VL lengthened intubation time compared to the Macintosh laryngoscope in patients with a difficult airway when performed by experienced operators [8].

The glottic view obtained using the McGrath VL was not superior to that obtained using the Macintosh laryngoscope in this study. VLs are generally known to offer an improved glottic view compared to direct laryngoscopes, which was confirmed for the McGrath VL in adults requiring tracheal intubation [8]. In addition, the McGrath VL provided better laryngeal views than the Macintosh laryngoscope in children with normal airways or in pediatric manikins with simulated difficult intubation [11,12,13]. Generally, this improvement in glottic view results from no need for alignment of the oral, pharyngeal, and laryngeal axes. However, the craniofacial and upper airway structures develop simultaneously [22], and congenital muscular torticollis is associated with asymmetrical cervical vertebrae, shoulders, ribs, and pulmonary system [2]. Thus, this musculoskeletal alteration may have negatively affected glottic visualization through the McGrath VL in our study.

There are two possible explanations for the extended intubation time and lack of improvement in glottic visualization when using the McGrath VL. First, successful tracheal intubation using VLs requires the following steps: (1) visualizing the airway and (2) inserting the endotracheal tube. Generally, VLs facilitate glottic visualization as a first step. However, this step may have been lengthened in our study. We used a conventional midline approach due to familiarity. However, this frequently resulted in the glottis not being visualized on the monitor because of a blind spot, thereby necessitating turning the scope to the left or right. Moreover, the reoriented scope affected the laryngologist’s hand–eye coordination and slowed the manipulation of the tracheal tube. Second, a manikin study showed that the space between the right side of the flange of the laryngoscope blade and the palatopharyngeal arch was narrower in the McGrath VL than in the Macintosh laryngoscope (8 mm vs. 15 mm) [23]. This may have contributed to the intubation outcomes in children with torticollis, even though it may not have had an effect in children with normal airways.

Our study had some limitations. First, the sample size was small; hence, the results cannot be generalized. Second, there is a possibility of substantial bias in the subjective parameters, including lifting force and difficulty level, as the laryngoscope was not blinded. Third, styletted tubes were used in both groups. Because stylets are not usually used for intubation using a Macintosh laryngoscope, the intubation time when using the Macintosh laryngoscope may have been overestimated considering the time required for removing the stylet. Fourth, we used the high-dose rocuronium. Since the dose of neuromuscular agent is an important factor in determining the quality of intubation, the depth of neuromuscular block may need to be considered in terms of lifting force [24]. Fifth, the age range of children in this study was too wide (12–107 months). Studies with separately enrolled infants, children, and school children may be needed. Lastly, further studies that consider the glottic view as the primary outcome with a large sample size may be needed to confirm the incidence of CL Grades III and IV in torticollis population and the improvement of glottic view in use of McGrath VL.

The difference of 5 s in the intubation time in our study may be small when considering pediatric difficult intubation and may have no clinical implications. However, this difference was observed when intubation was performed under elective and controlled conditions in the operating room by an experienced anesthesiologist. Hence, the intubation time could be longer in different clinical settings, e.g., in emergency conditions or in the intensive care unit and when performed by non-experts [25]. Although the higher IDS by limited neck mobility was more evident in the Macintosh group, our findings suggest that clinicians should choose the direct laryngoscope over the McGrath VL for airway management in children with torticollis.

## 5. Conclusions

Compared with the Macintosh laryngoscope, the McGrath VL extended the intubation time and did not improve glottic visualization, despite having a lesser lifting force, lower IDS, and similar success rate in children with torticollis. Further studies with larger sample sizes are needed to confirm these findings.

## Figures and Tables

**Table 1:** Intubation Difficulty Scale (IDS) calculation and scoring.

Parameters	Points
Number of attempts > 1	1 point each
Number of operators > 1	1 point each
Number of alternative techniques	1 point each
Cormack–Lehane grade minus 1	0 to 3 points
Operator perception of lifting force required	
Normal	0 point
Greater than in routine practice	1 point
Laryngeal pressure applied	
Not applied	0 point
Applied	1 point
Vocal cord mobility	
Abduction	0 point
Adduction and/or impeding tube passage	1 point
TOTAL IDS = SUM OF POINTS	

**Table 2:** Children’s characteristics, surgical findings, and airway assessment.

	McGrath (*n* = 15)	Macintosh (*n* = 15)	*p*-Value
Age, months	48.7 ± 33.2	42.5 ± 27.7	0.583
Gender, M/F	8/7	5/10	0.269
Height, cm	102.1 ± 20.5	97.4 ± 18.9	0.492
Weight, kg	16 [12.1–23 (10–28)]	15.6 [11.8–17.9 (8.5–41)]	0.619
ASA physical status 1	15 (100%)	15 (100%)	>0.999
Congenital muscular torticollis (right/left)	11/4	9/6	0.439
Surgical findings			
ROM rotation			
right, degree	53.7 ± 14.2	62 ± 17	0.156
left, degree	62.3 ± 16.8	65.3 ± 16.8	0.629
Lateral flexion			
right, degree	45 ± 14.8	43.3 ± 12.3	0.740
left, degree	38 ± 8.6	40.7 ± 13.3	0.521
**Fibrosis**			
SCM muscle—sternal head			0.605
(complete/partial/normal)	5/8/2	5/10/0	
(severe/moderate/mild)	7/4/4	5/9/1	
SCM muscle—clavicular head			0.142
(complete/partial/normal)	6/8/1	5/10/0	
(severe/moderate/mild)	10/3/2	6/9/0	
Preoperative airway assessment			0.847
Neck mobility (normal/reduced/fixed flexion)	5/9/1	7/7/1	
Loose upper incisors	1 (7%)	0	>0.999

Values are presented as mean ± standard deviation, median (interquartile range (range)) or number (%). ASA, American Society of Anesthesiologists; ROM, range of motion; SCM sternocleidmastoid muscle.

**Table 3:** Hemodynamic parameters.

	McGrath (*n* = 15)	Macintosh (*n* = 15)	*p*-Value
Heart rate (bpm)			
baseline	109 ± 18	108 ± 19	0.844
1 min after induction	110 ± 18	106 ± 21	0.560
before intubation	111 ± 18	108 ± 20	0.643
1 min after intubation	129 ± 15	123 ± 19	0.347
Mean blood pressure (mmHg)			
baseline	73 ± 17	73 ± 15	0.954
1 min after induction	63 ± 11	64 ± 13	0.880
before intubation	55 ± 7	58 ± 11	0.413
1 min after intubation	69 ± 11	63 ± 10	0.123

Values are presented as mean ± SD. Baseline means before induction.

**Table 4:** Intubation parameters.

	McGrath (*n* = 15)	Macintosh (*n* = 15)	*p*-Value
Intubation time, sec	31.4 ± 6.7	26.1 ± 5.4	0.025
Cormack–Lehane grade			0.101
1	9 (60%)	4 (27%)	
2	6 (40%)	8 (53%)	
3	0	3 (20%)	
OELM	3 (20%)	6 (40%)	0.427
Lifting force	0	10 (67%)	<0.001
IDS	1 [0–2 (0–2)]	2 [1–3 (0–5)]	0.022
Difficulty level			>0.999
Easy	8 (53%)	7 (47%)	
Moderate	6 (40%)	7 (47%)	
Difficult	1 (7%)	1 (7%)	
Intubation failure	2 (13%)	1 (7%)	>0.999

Values are presented as mean ± SD, median (interquartile range (range)) or number (%). OELM, optimal external laryngeal manipulation; IDS, intubation difficulty scale.

## Data Availability

Not applicable.
